# Magnitude of Podoconiosis and Its Associated Factors among an Adult Population in Waghmra Zone, Ethiopia: A Cross-Sectional Study

**DOI:** 10.1155/2020/9107562

**Published:** 2020-08-06

**Authors:** Addisu Getie, Tegene Atamenta, Ribka Nigatu, Aklilu Abera, Mingizem Girma

**Affiliations:** Department of Nursing, College of Health Sciences, Woldia University, Woldia, Ethiopia

## Abstract

**Background:**

Podoconiosis is one of the neglected tropical diseases, and it is a nonfilarial elephantiasis caused by prolonged barefoot exposure to irritant soil. This is manifested by slowly progressive swelling of the foot and lower leg which may lead to an irreversible disability among affected people. Podoconiosis is an entirely noncommunicable preventable disease with a low-cost preventive mechanism. This study is aimed at assessing the magnitude and factor affecting Podoconiosis in Waghmra zone, Amhara region, Ethiopia, 2019.

**Methods:**

A community-based cross-sectional study was conducted among 808 household members. A multistage sampling method was used to select study participants. A pretested semistructured questionnaire, observation, and clinical screening were used to collect data. The data were entered using EpiData version 4.2 and analyzed using SPSS version 24.0. Both bivariable and multivariable logistic regression models were fitted to identify factors associated with Podoconiosis. Odds ratio with 95% confidence interval was computed to determine the level of significance; in multivariable analysis, variables with a *P* value less than 0.05 were considered as statistically significant.

**Result:**

According this study's results, 4.3% of study participants were affected by Podoconiosis. A family number of four and above [AOR = 3.29, 95% CI (1.013-10.661)], family income of less than 500 ETB [AOR = 3.69, 95% CI (1.271-10.727)], distance from a health facility greater than 3 km [AOR = 6.69, 95% CI (1.610-27.863)], no shoe wearing habits [AOR = 5.02, 95% CI (1.969-12.778)], age at first wearing of shoes at 10 and above [AOR = 4.46, 95% CI (1.792-11.102)], and no feet washing habits [AOR = 3.87, 95% CI (1.516-9.883)] are significant factors of Podoconiosis.

**Conclusion:**

Creating awareness about the importance of shoe wearing, feet hygiene, and building infrastructure such as a road, health facility, and water supply were essential preventive strategies. Training about early diagnosis and effective management of lymphedema, giving emphasis on hygiene education and sanitation program, and reporting any suspected Podoconiosis case as early as possible are some of the recommendations forwarded.

## 1. Introduction

Podoconiosis is a chronic, debilitating neglected tropical inflammatory disease affecting genetically susceptible people who have prolonged contact with irritant red clay volcanic soils [1]. This prolonged exposure with irritant minerals from the soil may be due to working on the farm barefoot, and some individuals are exposed to irritants as a result of improper fit of the shoe with the foot. This has an impact on the fitness and functional efficiency of the feet. The foot is an important part of the human motor organ used for locomotion and is shaped uniquely in each individual. The shape and the construction of the foot have major impact on the quality of gait and postural stability. This state is conditioned by the proper capacity of muscles and ligaments and proper construction of the osteoarticular system [2]. Foot lesions can affect the distribution of loads on the plantar foot surface and thus lead to their deformation. The overall weakening of the bones, muscles, and ligaments increases the incidence of musculoskeletal deformities, not least those affecting the feet. Consequently, this leads to functional limitations, pain, and risk of falls [3].

Podoconiosis is one of the inflammatory diseases which affects the shape and function of the feet which leads to more soil exposure as a result of nonfitting shoes and pain during shoe wearing. It has different manifestations like itching of the skin of the forefoot and burning sensation in the foot and lower leg [1]. Podoconiosis currently does not have a specific treatment, but primary prevention consists of avoidance of prolonged contact between the skin and irritant soils by robust footwear or covering of floor surfaces in areas of irritant soil, training in foot hygiene like washing the legs daily with soap and water, and using antiseptics and emollients [4]. In 2013, eight priorities of the Neglected Tropical Diseases (NTDs) had been identified to eliminate and reduce the impact of these diseases by 2020. The strategy of morbidity management and disability prevention regarding Podoconiosis in Ethiopia is to increase the availability of care, to allow a flexible approach, to integrate care into other disease prevention programs, to provide quality of care, and to assure sustainability of care [5].

Worldwide, 4 million people are affected by Podoconiosis. It is also found in highland areas of tropical Africa, Central and South America, and different parts of Asia [6]. In Ethiopia, there are 345 districts which are endemic for Podoconiosis having a clay soil [7]. Podoconiosis has a wide range of impact, such as, a high economic and social burden; it is also associated with high stigmatization at school, workplace, or market as well as exclusion from social linkage like marriage [8].

Individuals who were affected by Podoconiosis lose around half of their total productive workdays. Direct and productivity costs of Podoconiosis in a group of 1.5 million inhabitants have been estimated at US$16 million a year, imposing an economic burden of $208 million per year in Ethiopia [9]. In Ethiopia, most people who have Podoconiosis experience complications other than economic and social impact. These include acute inflammation that may be aggravated by bacterial, viral, or fungal superinfection, at least once per year [10]. In northern Ethiopia, people with Podoconiosis were found to have a much lower quality of life scores, in all domains of quality of life like hygienic conditions, nutritional status, and accessibility of shoes [11]. So, these conditions cause one of the major complications of Podoconiosis which is acute adenolymphangitis. Acute adenolymphangitis is the major cause of morbidity in people with Podoconiosis which results in 149.5 days of activity lost per year [12].

Podoconiosis has a major impact on the human rights of patients. They are unable to realize their right to those necessities that are essential to reaching a standard of living that is adequate for health and well-being. Neglecting the health vulnerabilities eventually hampers social and economic opportunities. Podoconiosis has social, psychological, and economic burdens on patients and leads them to absenteeism and reduced working hours due to frequent acute attacks and fear of stigma. As a result, Podoconiosis has a considerable threat to education and employment opportunities [13]. People with Podoconiosis have constant pain and discomfort from the condition and smell offensively through chronic infection which is the primary cause of stigmatization [14]. Overall, Podoconiosis causes significant physical morbidity and devastating impact on mental health and quality of life and has catastrophic economic consequences for patients, families, and their community. Podoconiosis can be potentially eliminated, as the disease can be prevented by consistent use of footwear from early childhood, practicing proper foot hygiene, avoiding being barefoot in a farming area, accessing health services [15]. Even if Podoconiosis is preventable, different challenges deter prevention and treatment. These challenges include lack of awareness, little attention from government, lack of updated information on the prevalence of Podoconiosis, unavailability of prevention and treatment service centers, and different cultural practices. So, coordinated efforts should require supporting researches and developing health policy and educating and empowering those people living in endemic areas.

The purpose of this investigation is to identify the prevalence and associated factor of Podoconiosis in Waghmra zone, Amhara region, Ethiopia. The finding can be used to decrease the challenges of Podoconiosis prevention and treatment by increasing the awareness of individuals regarding shoe wearing habit, feet hygiene, early detection, and treatment.

## 2. Methods

### 2.1. Study Design and Setting

A community-based cross-sectional study design was conducted from March 1 to April 30, 2019, in Waghmra zone, north Ethiopia. Waghmra is a zone in the Amhara region of Ethiopia. Waghmra has a total population of 426,213 of whom 213,845 are men and 212,368 women.

### 2.2. Source Population

All people live in Waghmra zone are the source population.

### 2.3. Study Population

All people living in selected districts of Waghmra zone are the study population.

### 2.4. Inclusion Criteria and Exclusion Criteria

All people who live in the study area for at least six months were included in the study whereas those individuals who were unable to respond were excluded from this study.

### 2.5. Sample Size Determination

The sample size was calculated using a single population proportion formula designated as *n* = (*Z*2 *α*/2)*p*(1 − *p*) *d*2 based on the assumptions of *P* value = 0.054 which was the proportion of Podoconiosis [16], a 95% confidence level, 5% margin of error (*d*), 10% nonresponse rate, and design effect (DE) of 1.5. Accordingly, the total sample size calculated was 808.

### 2.6. Sampling Technique and Procedures

A multistage sampling technique was used. Waghmra zone has a total of 7 districts; from these, 3 were selected by using a simple random sampling method. Then, from each district, Kebeles were selected randomly and from each Kebele, households are selected randomly and proportionally allocated based on the total sample size. Lastly, the study subjects were selected by systematic random sampling technique from each household (Figure S1).

### 2.7. Data Collection Method

A structured interviewer-administered questionnaire was prepared and implemented after reviewing relevant literature. Data collection was conducted through face-to-face interviews, observable checklist, and clinical screening. The questionnaire was prepared in English and then translated to the local language (Amharic). To check its consistency, it was translated back to English.

### 2.8. Data Processing and Analysis

The data were entered using EpiData version 4.2 and exported to SPSS version 24 for analysis. Descriptive statistics like frequency, percentage, and standard deviation were computed. The binary logistic regression model was applied to identify determinant factors related to clinical practice competency. Variables with a *P* value of less than 0.2 on a bivariate analysis were entered into the multivariate analysis. A 95% confidence interval was used to identify associated factors in a multivariable binary logistic regression model. Hosmer–Lemeshow goodness of model fit was checked, and analysis was done by entering the procedure. Then, variables with a *P* value of less than 0.05 were considered as statistically significant.

## 3. Result

**Figure 1:**
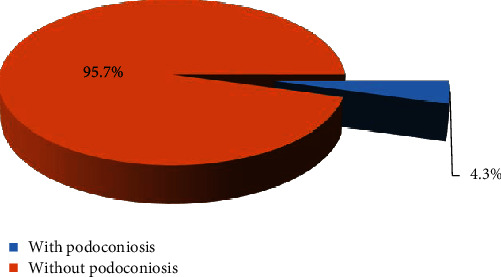
Proportion of Podoconiosis in Waghmra zone, Amhara region, Ethiopia, 2019.

This study revealed that the proportion of Podoconiosis is 4.3% (Figure 1). Of 792 participants, 43.4% were male and 56.6% were female. Half of the participants (49.2%) were found between the ages of 18 and 34 years and the mean (+SD) age was 36.01 (+11.160). Of the majority of participants, 96.1% were of Amhara ethnicity and 74.6% of participants were unable to read and write. Regarding family income, most of the participants (61.8%) were earning less than 500 ETB (Table S1). Regarding housing conditions, 69.4% of participants had nonpipe water sources (Table S2).

**Figure 2:**
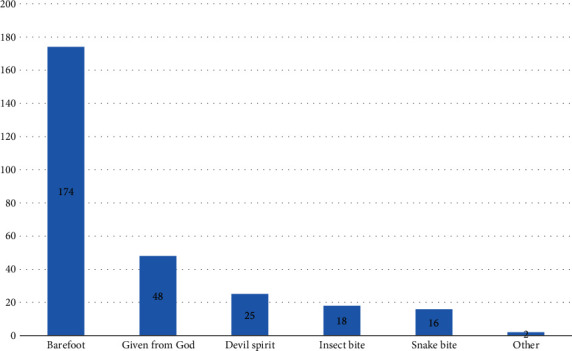
Cause of Podoconiosis mentioned by study participants in Waghmra zone, Amhara region, Ethiopia, 2019.

Among all diagnosed Podoconiosis patients, 41.2% were identified as stage two and 14.7% developed acute adenolymphangitis (Table S3). Of the 792 interviewed respondents, 663 (83.7%) wore shoes at the time of the interview and had shoe wearing habits. Of the 663 participants who had shoe wearing habits, 4.37% wore shoes only during holiday and 52.94% wore nonprotective shoes. Of the total participants, 33% wore shoes after the age of 10 years old. Concerning feet washing habits, the majority of study subjects (458 (57.8%)) had feet washing habits. Of the 458 participants who had feet washing habits, 91% were using only water for feet hygiene and 86.2% washed feet only once a day (Table S4). Out of all 792 study participants, 91 (11.5%) spent their time on different farming activities barefoot. From those 91 participants who spent time on farming activities barefoot, 65.9% spent more than 6.8 hours barefoot (Table S5). Around 44.8% of respondents were attending health education given by health extension workers and 30.1% have a radio or television (Table S6). Of the 792 participants, 450 (56.8%) did not hear about Podoconiosis and 283 (35.7%) answered the cause of Podoconiosis. From those participants who gave the answer about the cause of Podoconiosis, 174 (61.48%) of participants said that Podoconiosis is caused by being barefoot (Figure 2).

### 3.1. Factors Associated with Podoconiosis

Participants who had a current family number of four and above were found to be 3.29 more likely to develop Podoconiosis than those who had family numbers less than three [AOR = 3.29, 95% CI (1.01-10.66)]. Other variables which were significantly associated with Podoconiosis include family income less than 500 ETB [AOR = 3.69, 95% CI (1.27-10.73)], a distance of greater than 3 km from a health facility [AOR = 6.69, 95 CI (1.61-27.86)], absence of shoe wearing habits [AOR = 5.02, 95% CI (1.97-12.78)], and absence of feet washing habits [AOR = 3.87, 95% CI (1.52-9.88)] (Table 1).

## 4. Discussion

This community-based cross-sectional study assesses the magnitude of Podoconiosis and its associated factors among households in Waghmra zone, the Amhara region, in 2019. The result of this study showed that the prevalence of Podoconiosis is 4.3%. The finding of this study was less than the study done in Soddo Zuria district (5.4%), Midakgn district (7.4%), Bedele Zuria Werda (5.6%), and Ocholo village (5.1%) [12, 14, 17, 18]. The difference might be due to a general gradual increase in shoe wearing habits, accessibility of hygienic equipment like soap, and increasing awareness about overall social and hygienic factors. In contrast, the finding of this study was higher than the study done in Gulliso Erda (2.8%) and Wayu Tuka Wereda (3.05%) [19]. This difference might be due to lower foot washing and shoe wearing habits in the current study because the majority of participants were living in the rural area and subsequently walk barefoot during traveling for social services like marketing and farming. The odds of Podoconiosis was 3.29 times higher in a participant who had a current family number of four and above than those who had a family number less than three [AOR = 3.29, 95% CI (1.013-10.661)]. This might be because an increase in family size leads to financial problem; this may put a family in a difficult situation to buy shoes and other necessary hygienic materials like soap and water access. The odds of Podoconiosis was 3.69 times higher in a participant who had a family income less than 500 ETB as compared to a participant who had family income greater than 500 ETB [AOR = 3.69, 95% CI (1.271-10.727)]. This finding agrees with the study done in the East and West Gojjam zones [20]. This might be due to the reason that, as family income decreases, there are poor housing conditions, and so they were most likely barefoot and have difficult educational access, which lead to poor awareness about the prevention methods. The odds of Podoconiosis was 6.69 times higher in the study subject who was 3 km and above from the health facility than in the subject who was less than 3 km from the health facility [AOR = 6.69, 95% CI (1.610-27.863)]. This might be because an individual who is far from a health facility may lead to a decrease in health-seeking behavior. So, they do not have information about different preventive strategies and early detection of the disease. The study subjects who were barefoot were 5.02 times more likely to develop Podoconiosis than those who wore shoes [AOR = 5.02, 95% CI (1.969-12.778)]. This finding was similar to the study done in Soddo Zuria district, Midakegn district, East and West Gojjam zones, and tropical Africa [17, 18, 20, 21].

This might be because individuals who were barefoot for a prolonged period may have exposure to irritant minute mineral particles from the red clay soil, and this may lead to possible initiation of the pathophysiology of the disease. This leads to thickening and subsequent obstruction of the lymphatic system.

The other important predictor of Podoconiosis is the age when shoes are first worn. Those study subjects who wore their first shoes above 10 years were 4.46 times more likely to develop Podoconiosis than study subjects who wore their first shoes at 2-10 years [AOR = 4.46, 95% CI (1.792-11.102)]. This finding was inconsistent with the previous finding on Soddo Zuria district [20]. This might be because study subjects who wore shoes above 10 years may have prolonged time exposure to irritant red clay soil. Finally, our study showed that the odds of Podoconiosis in the study subject who did not have feet washing habits was 3.87 times higher than those who had feet washing habits [AOR = 3.87, 95% CI (1.516-9.883)]. This finding agrees with the study done in Midakegn district [18] and East and West Gojjam zones [20]. This might be because the feet washing practice may have a chance to remove dust or agents that trigger the disease process and prevent superinfection.

Even though Podoconiosis is an easily preventable and manageable disease, still, there is high prevalence. So, government organizations should plan modalities to enhance the shoe wearing and feet washing practices in line with the primary health care programs. These organizations should also address infrastructure such as road, health facility, water, and urbanization, building a Podoconiosis treatment center. Delivering a regular message on the importance of shoe wearing and feet hygiene, addressing shoe wearing coverage by distributing shoes free of charge, emphasis on hygiene education, and sanitation programs are core messages.

## 5. Conclusion

This study was conducted to assess the magnitude of Podoconiosis and its associated factors among households in Waghmra zone, Amhara region. Family number, distance from the health facility, shoe wearing habits, age of first shoe, and feet washing habits are a significant factors of Podoconiosis.

## Figures and Tables

**Table 1:** Factors affecting Podoconiosis in Waghmra zone, Amhara, Ethiopia, 2019.

Variable		Podoconiosis		COR (95% CI)	AOR (95% CI)	*P* value
		No	Yes			
Age	18-34	379	11	1	1	
	35-44	187	10	1.84 (0.77, 4.42)	1.6 (0.56, 4.55)	0.38
	45-64	192	13	2.33 (1.03, 5.31)	1.36 (0.5, 3.69)	0.55
Sex	Male	324	20	1	1	
	Female	434	14	0.52 (0.26, 1.05)	0.55 (0.22, 1.38)	0.2
Educational status	Illiterate	476	28	2.77 (1.13, 6.76)	1.68 (0.59, 4.81)	0.33
	Primary and above	282	6	1	1	
Occupation	Farming activities	434	26	2.43 (1.08, 5.43)	1.17 (0.41, 3.32)	0.77
	Nonfarming activities	324	8	1	1	
Family number	Less than 4	249	5	1	1	
	4 and above	509	29	2.84 (1.09, 7.42)	3.29 (1.01, 10.661)	0.048
Distance from health facility	Less than 3 km	672	17	1	1	
	3 km and above	86	17	7.81 (3.85, 15.87)	6.69 (1.61, 27.86)	0.009
Time taken to reach nearby health facility	Below mean (<22.07 km)	560	14	1	1	
	Mean and above (≥22.07 km)	198	20	4.04 (2.00, 8.15)	1.16 (0.3, 4.5)	0.83
Distance from water source	≤4 km	674	27	1	1	
	>4 km	84	7	2.08 (0.88, 4.92)	1.65 (0.52, 5.21)	0.4
Shoe wearing habit	Yes	648	15	1	1	
	No	110	19	7.46 (3.68, 15.12)	5.02 (1.97, 12.78)	0.001
Age at 1^st^ shoe wear	2-10 years	522	9	1	1	
	>10	236	25	6.14 (2.82, 13.37)	4.46 (1.79, 11.10)	0.001
Feet washing habit	Yes	449	9	1	1	
	No	309	25	4.04 (1.86, 8.77)	3.87 (1.52, 9.88)	0.005
Shoe wearing habit during traveling	Yes	647	20	1		
	No	111	14	4.08 (2.00, 8.32)	1.69 (0.61, 4.63)	0.31
Ever sick health facility	Yes	470	17	1	1	
	No	288	17	1.63 (0.82, 3.25)	2.51 (0.97, 6.51)	0.058

AOR: adjusted odds ratio; COR: crude odds ratio; CI: confidence interval.

## Data Availability

The data used to support the findings of this study are available from the corresponding author upon request.

## Abbreviations

AOR: Adjusted odds ratio
CI: Confidence interval
COR: Crude odds ratio
DS: Design effect
ETB: Ethiopian birr
km: Kilometer
NTDs: Neglected tropical diseases.

## Supplementary Materials

Supplementary Materials Table 1: sociodemographic characters of participants in Waghmra zone, Amhara region, Ethiopia, 2019. Table 2: distribution of housing condition of study participants in Waghmra zone, Amhara region, Ethiopia, 2019 Table 3: clinical features of Podoconiosis-diagnosed participants in Waghmra zone, Amhara region, Ethiopia, 2019. Table 4: behavioral characteristics of respondents in Waghmra zone, Amhara region, Ethiopia, 2019. Table 5: foot-soil exposure of respondents in Waghmra zone, Amhara region, Ethiopia, 2019. Table 6: exposure to media and health service utilization of respondents in Waghmra zone, Amhara region, Ethiopia, 2019.

## References

[1] World Health Organization (2018). Podoconiosis: endemic non filarial elephantiasis.

[2] Puszczałowska-Lizis E., Nowak K., Omorczyk J., Ambroży T., Bujas P., Nosiadek L. (2017). Foot Structure in Boys with Down Syndrome. *BioMed Research International*.

[3] Puszczalowska-Lizis E., Bujas P., Omorczyk J., Jandzis S., Zak M. (2017). Feet deformities are correlated with impaired balance and postural stability in seniors over 75. *PLoS One*.

[4] Tekola-Ayele F., Yeshanehe W. E. (2014). Podoconiosis: Tropical Lymphedema of the Lower Legs.

[5] Federal Democratic Republic of Ethiopia Ministry of Health (2016). Lymphatic filariasis and podoconiosis morbidity management and disability prevention guidelines.

[6] Davey G., Tekola F., Newport M. J. (2007). Podoconiosis: non-infectious geochemical elephantiasis. *Transactions of the Royal Society of Tropical Medicine and Hygiene*.

[7] Deribe K., Cano J., Giorgi E. (2017). Estimating the number of cases of podoconiosis in Ethiopia using geostatistical methods. *Wellcome Open Research*.

[8] Molla Y. B., Tomczyk S., Amberbir T., Tamiru A., Davey G. (2012). Patients’ perceptions of podoconiosis causes, prevention and consequences in east and west Gojam, Northern Ethiopia. *BMC Public Health*.

[9] Tekola F., Mariam D. H., Davey G. (2006). Economic costs of endemic non-filarial elephantiasis in Wolaita zone, Ethiopia. *Tropical Medicine & International Health*.

[10] Molla Y. B., Tomczyk S., Amberbir T., Tamiru A., Davey G. (2012). Podoconiosis in east and west Gojam zones, northern Ethiopia. *PLoS Neglected Tropical Diseases*.

[11] Mousley E., Deribe K., Tamiru A., Davey G. (2013). The impact of podoconiosis on quality of life in Northern Ethiopia. *Health Qual Life Outcomes*.

[12] Bekele K., Deribe K., Amberbir T., Tadele G., Davey G., Samuel A. (2016). Burden assessment of podoconiosis in Wayu Tuka woreda, east Wollega zone, western Ethiopia: a community-based cross-sectional study. *BMJ Open*.

[13] Shahvisi A., Meskele E., Davey G. (2018). A human right to shoes? Establishing rights and duties in the prevention and treatment of podoconiosis. *Health Human Rights*.

[14] Jagatai C. (2016). Neglected tropical disease - “podoconiosis”. *Journal of Pharmaceutical, Biological and Chemical Sciences*.

[15] Chandler D. J., Grijsen M. L., Fuller L. C. (2020). With bare feet in the soil: podoconiosis, a neglected cause of tropical lymphoedema. *Dermatology*.

[16] Ayelea F. T., Alemub G., Daveyc G., Ahrensd C. (2013). Community-based survey of podoconiosis in Bedele Zuria *woreda*, west Ethiopia. *International Health*.

[17] Alemtsehay E., Hana Y. (2016). Mengistu Podoconiosis prevalence and its associated factors in Soddo Zuria district, Wolayita zone South Ethiopia. *Journal of Pharmacy and Alternative Medicine*.

[18] Oli G. G., Ayele F. T., Petros B. (2012). Parasitological, serological and clinical evidence for high prevalence of podoconiosis (non-filarial elephantiasis) in Midakegn district, central Ethiopia. *Tropical Medicine & International Health*.

[19] Alemu G., Tekola Ayele F., Daniel T., Ahrens C., Davey G. (2011). Burden of podoconiosis in poor rural communities in Gulliso *woreda*, West Ethiopia. *PLoS Neglected Tropical Diseases*.

[20] Feleke B. E. (2017). Determinants of podoconiosis: a case control study. *Ethiopian Journal of Health Sciences*.

[21] Price E., Bailey D. (1984). Environmental factors in the etiology of endemic elephantiasis of the lower legs in tropical Africa. *Tropical and Geographical Medicine*.

