# Supplementary material for: Magnitude of Podoconiosis and Its Associated Factors among an Adult Population in Waghmra Zone, Ethiopia: A Cross-Sectional Study
Source: Biomed Res Int. 2020 Aug 6;2020:9107562. doi: 10.1155/2020/9107562 (PMC7428830; doi:10.1155/2020/9107562)
Supplement: Supplementary Materials — Table 1: sociodemographic characters of participants in Waghmra zone, Amhara region, Ethiopia, 2019. Table 2: distribution of housing condition of study participants in Waghmra zone, Amhara region, Ethiopia, 2019 Table 3: clinical features of Podoconiosis-diagnosed participants in Waghmra zone, Amhara region, Ethiopia, 2019. Table 4: behavioral characteristics of respondents in Waghmra zone, Amhara region, Ethiopia, 2019. Table 5: foot-soil exposure of respondents in Waghmra zone, Amhara region, Ethiopia, 2019. Table 6: exposure to media and health service utilization of respondents in Waghmra zone, Amhara region, Ethiopia, 2019. [file 9107562.f1.docx]

**Table 1:** Socio-demographic characters of participant in Waghmra zone, Amhara region, Ethiopia, 2019

| **Variables** | | **Frequency** | **Percent** |
| --- | --- | --- | --- |
| Age | 18-34 | 390 | 49.2 |
|  | 35-44 | 197 | 24.9 |
|  | 45-64 | 205 | 25.9 |
|  | Total | 792 | 100 |
| Sex | Male | 344 | 43.4 |
|  | Female | 448 | 56.6 |
|  | Total | 792 | 100 |
| Marital status | Single | 168 | 21.2 |
|  | Married | 519 | 65.5 |
|  | Divorced | 75 | 9.5 |
|  | Widowed | 30 | 3.8 |
| Religion | Orthodox | 741 | 93.6 |
|  | Muslim | 50 | 6.3 |
|  | Protestant | 1 | 0.1 |
| Ethnicity | Amhara | 761 | 96.1 |
|  | Tigray | 29 | 3.7 |
|  | Oromo | 2 | 0.3 |
| Educational status | No education | 504 | 63.6 |
|  | Primary | 207 | 26.1 |
|  | Secondary | 56 | 7.1 |
|  | Collage and Above | 25 | 3.2 |
| Occupational status | Farmer | 460 | 58.1 |
|  | House wife | 117 | 14.8 |
|  | Merchant | 72 | 9.1 |
|  | Governmental employed | 41 | 5.2 |
|  | Private employed | 34 | 4.3 |
|  | Others | 68 | 8.6 |
| Family size | One | 22 | 2.8 |
|  | Two to three | 232 | 29.3 |
|  | Four and above | 538 | 67.9 |
| Family income | <500 | 487 | 61.8 |
|  | 501-800 | 67 | 8.5 |
|  | 801-1500 | 131 | 16.5 |
|  | >1500 | 107 | 13.5 |
| Distance from health facility | < 3 km | 689 | 87 |
|  | 3 and above km | 103 | 13 |
| Time taken to reach health facility | Bellow mean (22.07 min) | 574 | 72.5 |
|  | Mean and above (>=22.07 min) | 218 | 27.5 |
| Distance from water resource | <=4km | 701 | 88.5 |
|  | 5-9 km | 72 | 9.1 |
|  | Greater than 9 | 19 | 2.4 |
| Time taken to reach water resource | Less than mean (23.81 min) | 534 | 67.4 |
|  | Mean and above (>=23.81 min) | 258 | 32.6 |

**Table 2:** Distribution of housing condition of study participant in Waghmra zone, Amhara region, Ethiopia, 2019

| **Variable** | | **Frequency** | **Percentage** |  |
| --- | --- | --- | --- | --- |
| Water source | Pip | 242 | 30.6 |  |
|  | Non pip | 550 | 69.4 |  |
| Type of floor | Cement(wood) | 11 | 1.4 |  |
|  | Earth | 781 | 98.6 |  |
| Type of wall | Cement | 23 | 2.9 | |
|  | Mud | 769 | 97.1 |  |
| Type of roof | Concrete | 41 | 5.2 |  |
|  | Iron sheet | 646 | 81.6 |  |
|  | Other | 105 | 13.3 |  |

**Table 3:** Clinical features of Podoconiosis diagnosed participant in Waghmra zone, Amhara region, Ethiopia 2019

| **Variable** | | **Frequency** | **Percentage** |
| --- | --- | --- | --- |
| Podoconiosis stage | Stage one | 13 | 38.2 |
|  | Stage two | 14 | 41.2 |
|  | Stage three | 7 | 20.6 |
|  | Stage four | 0 | 0 |
|  | Stage five | 0 | 0 |
|  | Total | 34 | 100 |
| Podoconiosis complication | With acute Adenolymphangitis | 5 | 14.7 |
|  | Without acute Adenolymphangitis | 29 | 85.3 |
|  | Total | 34 | 100 |

**Table 4:** Behavioral characteristics of respondents in Waghmra zone, Amhara region, Ethiopia 2019

| **Variable** | | **Frequency** | **Percentage** |
| --- | --- | --- | --- |
| Shoe wearing habit | Yes | 663 | 83.7 |
|  | No | 129 | 16.3 |
| Time of wearing shoe | holy day | 29 | 4.37 |
|  | Weekends | 18 | 2.71 |
|  | Rarely | 77 | 11.61 |
|  | Always | 539 | 81.31 |
|  | Total | 663 | 100 |
| Shoe wearing at the time of interview | Yes | 663 | 83.7 |
|  | No | 129 | 16.3 |
|  | Total | 663 | 100 |
| Character of shoes | Protective | 312 | 47.06 |
|  | Not protective | 351 | 52.94 |
|  | Total | 663 | 100 |
| Average age of first shoes | 2-10 years | 531 | 67 |
|  | >10 years and not at all | 261 | 33 |
|  | Total | 792 | 100 |
| Substitution time of old shoes | Being old | 530 | 79.94 |
|  | Before old | 88 | 13.27 |
|  | As needed | 45 | 6.79 |
|  | Total | 663 | 100 |
| Feet washing habit | Yes | 458 | 57.8 |
|  | No | 334 | 42.2 |
| Feet washing materials | Water only | 417 | 91 |
|  | Water and soap | 41 | 9 |
|  | Total | 458 | 100 |
| Feet washing per day | One times | 395 | 86.2 |
|  | Two times | 60 | 13.1 |
|  | Three times | 3 | 0.7 |
|  | Total | 458 | 100 |

**Table 5:** Foot-soil exposure of respondents in Waghmra zone, Amhara region, Ethiopia 2019

| **Variable** | | **Frequency** | **Percentage** |
| --- | --- | --- | --- |
| Shoe wearing habit during travelling for social purpose | Yes | 665 | 83.96 |
|  | No | 127 | 16.04 |
|  | Total | 792 | 100 |
| Shoe wearing habit during faming activity | Yes | 701 | 88.5 |
|  | No | 91 | 11.5 |
|  | Total | 792 | 100 |
| Time spent in farming activities on bar foot | Bellow mean hr. (<6.84 hr.) | 31 | 34.1 |
|  | Mean and above hr. (>=6.84 hr.) | 60 | 65.9 |
|  | Total | 91 | 100 |

**Table 6:** Exposure to media and health service utilization of respondents in Waghmra zone, Amhara region, Ethiopia 2019

| **Variable** | | **Frequency** | **Percentage** |
| --- | --- | --- | --- |
| Having radio or TV | Yes | 238 | 30.1 |
|  | No | 554 | 69.9 |
| Attending radio or TV | Yes | 229 | 28.9 |
|  | No | 563 | 71.1 |
| Time of attending radio or TV | Sometimes | 47 | 20.5 |
|  | Weekly | 85 | 37.1 |
|  | Every day | 63 | 27.5 |
|  | As needed | 34 | 14.8 |
|  | Total | 229 | 100 |
| Attending health education program | Attend | 89 | 11.2 |
|  | No attend | 703 | 88.8 |
| Ever heard about Podoconiosis | Yes | 29 | 3.7 |
|  | No | 763 | 96.3 |
| Ever seek health facility | Yes | 487 | 61.5 |
|  | No | 305 | 38.5 |
| If yes type of health service | For MCH service | 90 | 18.48 |
|  | For investigation | 135 | 27.72 |
|  | Because I am seek | 262 | 53.80 |
|  | Total | 487 | 100 |
| Attending health education | Yes | 355 | 44.8 |
|  | No | 437 | 55.2 |

*****TV= Television

Waghmra zone (7 Werdas)

Dehana Werda (31 kebeles)

Zikula Werda (15 kebeles)

Sehala Werda (9 kebeles)

| Amed | 1481 HH | 114HH |
| --- | --- | --- |
| Amed zuria | 1401 HH | 108 HH |
| Yeshewa | 950 HH | 74 HH |
| Ayin Mariam | 885 HH | 68 HH |
| Tserwala | 702 HH | 54 HH |
| Tsamola | 786 HH | 60 HH |

| Tsetseka | 1667 HH | 128HH |
| --- | --- | --- |
| Netsant | 707 HH | 54HH |
| Deber hiwot | 1093HH | 84HH |

| Akegn | 484 HH | 37 HH |
| --- | --- | --- |
| Deber selam | 355 HH | 27 HH |

**n=808**

HH= Household

Figure 1: Sampling procedure of respondents in Waghmra zone, Amhara region, Ethiopia 2019
